# Dual-Drug Delivery via Zein In Situ Forming Implants Augmented with Titanium-Doped Bioactive Glass for Bone Regeneration: Preparation, In Vitro Characterization, and In Vivo Evaluation

**DOI:** 10.3390/pharmaceutics14020274

**Published:** 2022-01-24

**Authors:** Alaa Emad Eldeeb, Salwa Salah, Mostafa Mabrouk, Mohammed S. Amer, Nermeen A. Elkasabgy

**Affiliations:** 1 Department of Pharmaceutics and Industrial Pharmacy, Faculty of Pharmacy, Cairo University, Kasr El-Aini Street, Cairo 11562, Egypt; Salwa.salah@pharma.cu.edu.eg (S.S.); Nermeen.ahmed.elkasabgy@pharma.cu.edu.eg (N.A.E.); 2 Refractories, Ceramics and Building Materials Department, National Research Centre, Giza 12622, Egypt; Mostafamabrouk.nrc@gmail.com; 3 Department of Surgery, Anaesthesiology and Radiology, Faculty of Veterinary Medicine, Cairo University, Cairo 12211, Egypt; Mohammedvet@cu.edu.eg

**Keywords:** dual-medicated implant, zein protein, pitavastatin, tedizolid, bioactive glass, bone tissue engineering

## Abstract

In situ forming implants (IFIs) are non-surgical approach using biodegradable polymers to treat bone fractures. The study aimed at preparing dual-drug-loaded IFIs to deliver pitavastatin (osteogenic drug) and tedizolid (antibiotic) using zein as the implant matrix via solvent-induced phase inversion method. At first, several investigations were done on pitavastatin-loaded zein IFIs, where three concentrations of zein were used (10, 20, and 30% *w/v*). IFIs were evaluated for their solidification time, rheological properties, injectability, and in vitro release. IFIs containing bioactive glass nanoparticles were prepared by the addition of non-doped bioactive glass nanoparticles (BGT_0_; 1, 3, 5, and 10% *w/v*) or titanium-doped bioactive glass nanoparticles (BGT_5_; 1% *w/v*) to the selected concentration of zein (30% *w/v*) and then evaluated. The optimized dual-medicated implant (D-ZIFI 1) containing pitavastatin, tedizolid, sodium hyaluronate (3% *w/v*), and BGT_5_ (1% *w/v*) was prepared and compared to IFI lacking both sodium hyaluronate and BGT_5_ (D-ZIFI 2). D-ZIFI 1 and 2 sustained the release profiles of both drugs for 28 days. SEM images proved the interconnected porous structure of D-ZIFI 1 due to sodium hyaluronate. In vivo studies on surgically induced bone defects in Sprague–Dawley rats signified the proper accelerated bone healing ability of D-ZIFI 1 over D-ZIFI 2. Results presented D-ZIFI 1 as a promising, effective, non-surgical approach for bone healing.

## 1. Introduction

Bone diseases resulting from osteoporosis, trauma, malignancy, and other reasons represent about half of the chronic diseases in patients over 50 years old, necessitating in some cases surgical interventions that impose a growing burden on the health-care sector [1]. Bone defects could be categorized into non-critical- and critical-sized defects [2]. Non-critical-sized defects could be repaired by the normal bone regeneration process, while the critical-sized ones do not heal normally and are considered as a serious problem requiring surgical intervention to replace the damaged bone tissues at the site of the defect by grafting an external bone tissue [3,4]. Tissue replacement could be done by autografting (transplanting tissues from the same injured individual) or allografting (transplanting from one individual to another). However, these two approaches are costly and there is a high susceptibility to donor site morbidity, hematoma, and infection in addition to the rejection of the graft that may occur [5]. Xenografts (transplanting from a donor of a different species) show complications similar to allografts [5]. Metallic implants made of stainless steel or cobalt, for example, are also used for grafting, but they possess significant limitations; they lead to stress shielding (the reduction in bone density, or osteopenia, as a result of the removal of typical stress from the bone by an implant), and repeated surgical interventions are required [3,6].

Bone tissue engineering is considered a radical and hopeful approach for repairing bone defects as it depends on tissue regeneration rather than tissue replacement [7]. A variety of biomaterials derived from natural or synthetic polymers and bioactive glasses have been used over the past few decades to generate different forms of 3D networks, such as hydrogels, fibers, and implants, besides a wide variety of drug delivery systems [8]. Among the designed drug delivery systems, in situ forming implants (IFIs) provide an attractive potential for the controlled treatment of bone defects. IFIs are liquid formulations injected at the site of the defect, where they solidify. IFIs possess several advantages over traditional implantation procedures, being less invasive and having the ability to adapt to the geometry and size of the targeted defect [9]. The ideal scaffold should (i) be biocompatible, to be safely incorporated into the host tissues, (ii) be biodegradable, where the scaffold degradation occurs parallel to the formation of the new tissues, so no surgical removal of the scaffolds is required, (iii) be porous, to allow the transportation of new cells as well as the formation of new tissues within the scaffold, (iv) have enough mechanical strength to provide temporary skeletal support, and (v) have minimal antigenicity [10].

Several biomaterials are widely used in the tissue engineering field, such as chitosan [11], nanofibrillated cellulose [12], alginates [13], and proteins [14]. Protein-based biomaterials have recently gained much interest in the tissue engineering field as they exhibit better biocompatibility when compared with synthetic polymers due to their similarity with the natural proteins present in the extracellular matrix of bones [15]. Plant-based proteins show superiority over animal-based ones as they are easier to obtain and have less potential to elucidate an immunologic response in the host tissues [16]. Zein is a high-molecular-weight plant protein (22–29 kDa) that belongs to the prolamins class. Zein is an inexpensive, biodegradable, and biocompatible protein that is classified as generally regarded as safe biomaterial (GRAS) by the FDA [17]. Zein is mainly composed of hydrophobic amino acids, which give it a characteristic hydrophobicity, hence making it an excellent skeleton material to provide sustained drug release as the spiral structure of it allows ample amounts of the drug to be loaded [17]. These properties make zein an excellent candidate in the tissue engineering field [14,18]. Additionally, the growing potential of the specific use of zein in bone tissue engineering is due to its perfect biocompatibility, biodegradability, suitable mechanical properties, good resistance to microbial degradation, and inherent anti-oxidant activity [19,20]. Zein has been widely used in bone rejuvenation in various forms, including membranes [21], scaffolds [22], microspheres [23], and nanofibers [24]. However, its ability to stimulate cell proliferation is limited, so the combination of bioactive substances (e.g., calcium phosphates, bioactive glass, and osteogenic drugs) with zein is advisable to provide the means to induce osteoblastic differentiation and proliferation [25,26].

Bioactive glass (BG) nanoparticles is a term that defines itself by the name; “bioactive” refers to the ability of a substance to elicit a biological response at the interface of the material, resulting in the formation of a specific bond between the material and the surrounding tissue [27]. BG is a biocompatible, biodegradable substance related to bioceramics and is widely used in orthopedic clinical applications [28].

Pitavastatin is a statin drug that is used for lowering blood cholesterol levels by inhibiting hydroxymethylglutaryl coenzyme A reductase, the key enzyme in the mevalonate pathway for cholesterol biosynthesis. Moreover, it has been reported that pitavastatin has a stimulatory effect on bone formation through more than one mechanism. Pitavastatin enhances osteoblastic activity by stimulating bone morphogenetic proteins-2 (BMP-2), which are identified as the osteoinductive components in bones [29]. Pitavastatin also promotes the expression of osteocalcin; one of the major non-collagenous proteins of the bone secreted by osteoblasts, it influences matrix mineralization and is regarded as a marker for bone formation [30,31,32,33]. Furthermore and based on its antihypercholesteremic effect, it was stated that inhibiting the mevalonate pathway blocks, in turn, the formation of prenylated proteins, which are necessary for osteoclast function and survival [34].

This research aimed to explore the best conditions for fabricating biodegradable, biocompatible, and bioactive IFIs using zein as the implant forming material and applying a solvent-induced phase inversion method. BG was added to enhance the osteogenic abilities of the formed implants. The optimized formulation was loaded with sodium hyaluronate as a porogenic material to improve cell proliferation. For enhancing the therapeutic usefulness of the prepared implants, multi-active IFIs combining two active ingredients, pitavastatin and tedizolid, were prepared. Tedizolid is an antibiotic added to treat the infections associated with bone injuries. Ease of injectability, rate of solidification, rheological behavior, as well as the in vitro release of the prepared IFIs were studied, followed by morphological examination and compatibility testing. Finally, the therapeutic efficacy of the implant was evaluated in vivo on Sprague–Dawley rats with surgically induced bone injuries.

## 2. Materials and Methods

### 2.1. Materials

Pitavastatin calcium was kindly supplied by Hochster Pharmaceutical Industries (Cairo, Egypt). Tedizolid phosphate was donated by Hikma Pharmaceuticals (Cairo, Egypt). Zein protein (molecular weight 22–27 KDa) and sodium hyaluronate (molecular weight 90–100 KDa and viscosity 0.74–2.38 cp) were purchased from Acros Organics (Belgium). Dimethyl sulfoxide (DMSO) was procured from Loba Chemie, India. Dialysis membrane with a molecular-weight cut-off of 12,000–14,000 g/mol was brought from Sigma-Aldrich (Darmstadt, Germany). Betolvex^®^ ampoules (batch number KDE0738) were manufactured by Minapharm Pharmaceutical and Chemical Industries (10th of Ramadan, Egypt). All other reagents were of analytical grade and used as received. All water used was distilled, deionized water.

### 2.2. Preparation of Zein In Situ Forming Implants (ZIFIs)

ZIFIs were formulated by solvent-induced phase inversion method [35]. Briefly, accurately weighed amounts of pitavastatin (5 mg/mL) and zein were dissolved in 1 mL of DMSO in a screw-capped glass vial using a water bath sonicator (Crest Ultrasonic Corp., Trenton, NJ, USA) until complete dissolution. Different concentrations of zein were tried; 10, 20, and 30% *w/v*. The composition of the prepared implants is represented in Table 1.

### 2.3. In Vitro Characterization of the Prepared Zein In Situ Forming Implants (ZIFIs)

#### 2.3.1. In Vitro Solidification Time

**Figure 1:**
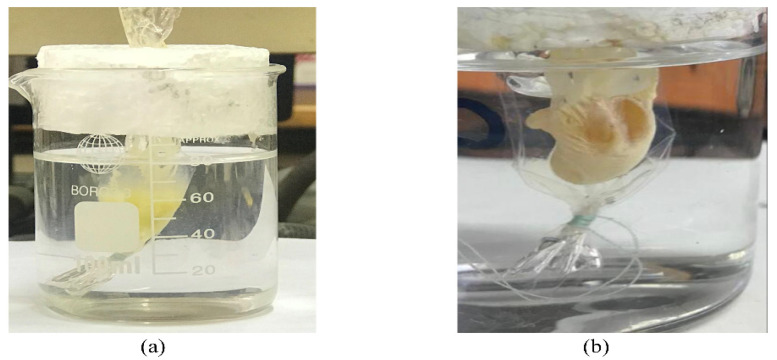
Brief representation of solidification time test; (**a**) during the solidification of the in situ forming implant and (**b**) after the solidification of the implant.

The in vitro solidification time of the prepared formulations was determined by the dialysis membrane/solidification time technique [36]. As shown in Figure 1a, a 6 cm dialysis membrane was immersed in a 100 mL beaker containing 80 mL of phosphate buffer saline (PBS; pH 7.4) and kept at 37 °C to mimic the body temperature. The dialysis membrane was clamped at its lower side with a string and remained suspended in the beaker with its upper side fixed by a piece of foam. Then, 1 mL of each formulation was introduced into a dialysis membrane pre-filled with 1.5 mL of PBS. The solidification time was assessed by recording the time taken by the investigated formulation to completely solidify [36], as presented in Figure 1b.

#### 2.3.2. Rheological Properties

Rheological characteristics of the formulations were determined using the cone and plate rheometer (Brookfield DV3THB cone/plate rheometer, spindle CPE-40, and RheocalcT software, v1.1.13, Middleboro, MA, USA). The temperature was kept constant at 25 ± 2 °C using a water bath (PolyScience model 9006, Niles, IL, USA) encircling the external cylinder during the operation. A volume of 1 mL of the examined formulation was placed on the plate of the rheometer, and the speed setting of the rheometer was altered within 20 s between every two successive speeds (ranging from 10 to 250 rpm). The flow behavior of the investigated samples was determined by plotting the log of the shear stress values (log *S*) versus the log of the shear rate values (log *D*) as per Farrow’s equation [37]:

log D=NlogS−log η

where *D* and *S* represent the shear rate (s^−1^) and the shear stress (Pa), respectively; *N* presents Farrow’s constant; and η is viscosity (Pa.s).

According to Farrow’s constant (*N*) values, the type of flow could be determined. *N* values equal to 1 specify Newtonian flow, whereas values less than or more than 1 indicate shear-thickening and shear-thinning behaviors, respectively.

#### 2.3.3. Injectability Test

To assess the ease of injectability, the flow rate of the prepared formulations was determined and compared to Betolvex^®^ employing designed equipment similar to that formerly described by Leroux et al. [38] but with some modifications, where a 3 mL syringe attached to a 19-gauge needle was bound to a rubbery tube linked to an air pump. A constant volume of the tested formulations (1 mL) was added to the syringe, and then the air was ejected from the air pump to the surface of the solution (at a constant pressure of 70 mmHg measured by sphygmomanometer). The time taken for the release of the formulation from the syringe was determined and the flow rate (mL/min) values were calculated [39].

#### 2.3.4. In Vitro Release Study

The dialysis bag diffusion technique was employed to determine the in vitro release pattern of the formed implants [36]. Dialysis bags were soaked in distilled water overnight. Then, one end of each bag was clamped. The bags were filled with 1.5 mL of PBS followed by the injection of a precisely measured volume (1 mL) of the tested formulation. The bags were then secured by clamps on the other end. At that time, the secured dialysis bags were immersed in glass bottles pre-filled with 20 mL of PBS (pH 7.4). The bottles were kept in a thermostatically controlled shaking water bath (Witeg, Wertheim, Germany) at 100 rpm and 37 ± 0.5 °C. Different sampling points were investigated, first at 1, 2, 4, 6, 8, and 24 h and then at 4, 7, 14, 21, and 28 days. At each sampling point, the whole medium was replaced with a fresh release medium. The percentage of pitavastatin released at each time point was calculated by determining the UV absorbance (Shimadzu, model UV-1800 PC, Kyoto, Japan) of each sample at λ_max_ 244 nm after suitable dilution. The percentages of drug released after 24 h (Q_24h_) were compared to detect the burst drug release. The release of pitavastatin from its PBS suspension was achieved following the same procedures to ensure that the dialysis membrane was suitable for the study.

The release data were fitted into Korsmeyer–Peppas models [40,41], and the release rate constant (k) for each formula was calculated and compared.

### 2.4. Preparation and Evaluation of Zein In Situ Forming Implants Loaded with Bioactive Glass Nanoparticles (BG-ZIFI)

#### 2.4.1. Preparation of Bioactive Glass (BG) Nanoparticles

A combination of distilled water and ethyl alcohol in the ratio 1:1 (total volume 600 mL) was used for the hydrolysis of a pre-determined volume of tetraethyl orthosilicate that was kept under acidic conditions (using drops of concentrated hydrochloric acid). The mixture was stirred for 30 min before calcium nitrate tetrahydrate was added, which was followed by stirring for another 30 min. Afterward, triethyl phosphate was added to the formed solution and the blend was mixed at room temperature for an additional 30 min or until the blend became homogenous and transparent. Titanium doping was carried out following the same procedures, and then titanium isopropyl (C_12_H_28_O_4_Ti) was added as a precursor for TiO_2._ A gel was obtained that was then placed in a furnace at 70 °C overnight to be dried. Further calcination was performed at 550 °C for 2 h [42]. The composition (% w/w) of the non-doped bioactive glass nanoparticles (BGT_0_) was 45% CaO, 5% P_2_O_5_, and 50% SiO_2_ while that of the titanium-doped bioactive glass nanoparticles (BGT_5_) was 40% CaO, 5% P_2_O_5_, 50% SiO_2_, and 5% TiO_2_. The prepared systems proved to be glassy in nature as they exhibited glass transition temperatures in the range of 500–580 °C, as confirmed by their DSC analyses. In addition, they exhibited amorphous XRD patterns in the absence and existence of a titanium dopant, as demonstrated in a previous research work [42].

#### 2.4.2. Evaluation of Bioactive Glass Nanoparticles by Transmission Electron Microscope (TEM)

A high-resolution TEM (AJEM 2100, JEOL, South Dakota, Japan) was used to visualize the morphologies and particle sizes of BGT_0_ and BGT_5_. The samples were properly diluted and dispersed in distilled water and then adsorbed on a copper grid coated with carbon. A filter paper was employed to remove the extra dispersion. The sample was then dried for 15 min before examination at room temperature [43].

#### 2.4.3. Loading Zein In Situ Forming Implants with Bioactive Glass Nanoparticles

ZIFIs filled with bioactive glass nanoparticles (BG-ZIFI) were prepared following the same procedures as mentioned in Section 2.2 but with a slight modification, where the desired concentration of BG was dispersed in the homogenous drug-in-zein organic solution by vortexing for 2 min. Four concentrations of non-doped BG (BGT_0_; 1, 3, 5, and 10% *w/v*) as well as one concentration of titanium-doped BG were used (BGT_5;_ 1% *w/v*). The comprehensive composition of the formed IFIs is displayed in Table 1.

#### 2.4.4. In Vitro Release Study

The effect of the addition of various concentrations of BGT_0_ and BGT_5_ on the in vitro release behavior of the modified IFIs was evaluated following the same procedures as mentioned in Section 2.3.4.

### 2.5. Preparation of Dual-Medicated Zein In Situ Forming Implants (D-ZIFI)

Tedizolid phosphate was added to the selected formulation BG-ZIFI 5 consisting of 30% *w*/*v* zein and improved by the addition of 1% *w*/*v* BGT_5_. In brief, tedizolid (5mg/mL) was dissolved with pitavastatin in the organic solution of formulation BG-ZIFI 5 by vortexing. Sodium hyaluronate (3% *w*/*v*; sieved through a 500 µm sieve) was dispersed in the drug-in-zein solution by vortexing after dissolving all the implant components (both drugs, zein, besides dispersing BGT_5_). The optimized dual-medicated ZIFI was coded as D-ZIFI 1. For comparison purposes in the animal study, dual-medicated ZIFI lacking the addition of both BGT_5_ and sodium hyaluronate was prepared (D-ZIFI 2; composed of 30% zein, pitavastatin, and tedizolid). The composition of dual-medicated implants is presented in Table 1.

### 2.6. Characterization of Dual-Medicated Zein In Situ Forming Implants (D-ZIFI) 

Time for in vitro solidification, rheological characteristics, and injectability checking were studied, as stated in Section 2.3.

#### 2.6.1. In Vitro Release Study

The in vitro release profiles as well as kinetic parameters of pitavastatin and tedizolid from the implants were determined by the dialysis bag diffusion technique as mentioned in Section 2.3.4 Samples from the release medium were analyzed applying the second derivative method [44] by measuring the amplitudes of samples at 271.6 (zero crossing of tedizolid) and 303 nm (zero crossing of pitavastatin), for the determination of pitavastatin and tedizolid, respectively.

#### 2.6.2. Fourier-Transform Infrared (FTIR) Spectroscopy

FTIR spectra for the selected formulations, their physical mixtures, and individual ingredients were determined at room temperature using FTIR-8400 (Shimadzu, Kyoto, Japan). About 3–4 mg of each sample was mixed with dry potassium bromide to be compressed in the form of a disc and then examined at the scanning range of 4000–400 cm^−1^.

#### 2.6.3. Differential Scanning Calorimetry (DSC)

DSC thermograms were determined using DSC (DSC-50, Shimadzu, Kyoto, Japan) for the chosen formulations, their physical mixtures, and their separate components. Samples of 2 mg were introduced to an aluminum pan with a flat bottom and then heated at a heating rate of 10 °C/min up to a temperature of 350 °C. The surrounding air was composed of inert nitrogen [45].

#### 2.6.4. Morphological Examination

The external and internal morphologies of the examined implants were investigated. Cross-sectional morphology of the selected implants was determined using Tescan scanning electron microscope (SEM; Tescan Vega, Czech Republic). Before the scanning step, the selected D-ZIFIs solutions were injected into 1.5 mL of PBS (pH 7.4), secured in a dialysis bag at 37 °C, and then added to glass bottles containing 20 mL of PBS (as the in vitro release study). Then, the formed implants were withdrawn from the release medium after 1 and 7 days and were air-dried overnight on a filter paper. Dried samples were secured on stubs using double-faced adhesive tape, followed by sputter-coating with a thin layer of gold. Imaging was performed at 20 kV.

#### 2.6.5. Effect of Gamma Sterilization

Gamma irradiation evolving from a ^60^Co irradiator (Gamma cell 1000; BEST Theratronics, Ontario, Canada) at a 20 kGy irradiation dose was used to sterilize the formulations [46]. The sterilized formulations were re-evaluated for their in vitro solidification time, injectability, and in vitro release following the same steps as for the non-sterilized formulations. The release profiles of the selected formulations before and after sterilization were compared by calculating the similarity factor (*f*_2_) applying this equation [47]:

$$f_{2}=50\;\log_{10}\left\{{\left[1+\left(\frac{1}{n}\right)\overset{n}{\underset{i=1}{\sum }}{\left(R_{t}-T_{t}\right)}^{2}\right]}^{-0.5}\times 100\right\}$$

where *n* is the number of sampling points and *R* and *T* are the percentages of the drug released from the same tested sample prior to and post sterilization, respectively, at time *t*.

### 2.7. Statistical Analysis

Data are expressed as the mean ± standard deviation. The results were statistically analyzed by SPSS^®^ software, version 25 (SPSS Inc., IBM Corporation, New York, NY, USA) using one-way ANOVA. Then the least significant difference (LSD) test was selected as a post hoc test. The statistical level of significance in all experiments was set at *p*-value < 0.05.

### 2.8. In Vivo Animal Study

#### 2.8.1. Animals

The protocol of the research was reviewed and authorized by the Research Ethics Committee in the Faculty of Pharmacy, Cairo University, Egypt (PI-2678). The applied procedures were in accordance with the Guide for the Care and Use of Laboratory Animals of the National Institutes of Health (NIH publication No. 85–23, 1996). Thirty-two male Sprague–Dawley rats weighing 200–250 g were used in the research. The rats were isolated, four rats each, and were kept in separate cages at the animal house at the Faculty of Veterinary Medicine, Cairo University, Egypt. The animals were fed with standard food and water. The cages were kept in air-conditioned rooms at 25 ± 0.5 °C and lit with artificial fluorescent light for 12 h and kept in the dark for 12 h to attain alternating cycles of day and night. The animals were randomly divided into two equal groups: group I (D-ZIFI 1) and group II (D-ZIFI 2). The animals of each group were subjected to an induced proximal tibial defect (1 mm in diameter) in both hind limbs by a surgery followed by the injection of the selected formulation in the defect. The defect area was observed at 2, 4, 6, and 8 weeks post-injection.

#### 2.8.2. Surgical Procedures

**Figure 2:**
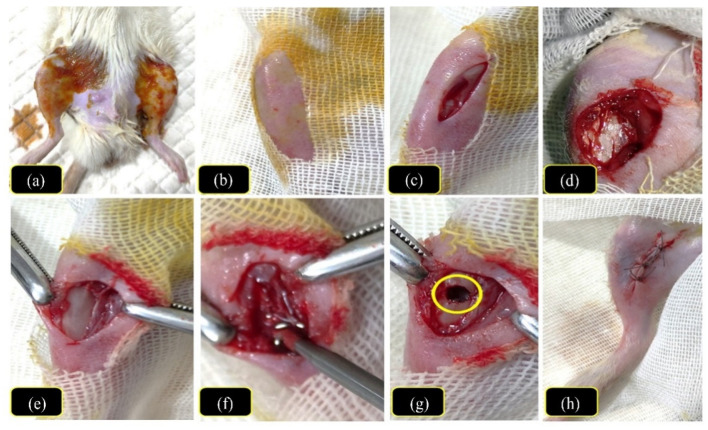
The photographs are showing the steps of the surgical procedures of the induced tibial bone defect; (**a**) the hind limbs were shaved and prepared aseptically, (**b**) the exposed tibial region, (**c**) the skin incision made at the flat part of the tibial shaft, (**d**) cutting the subcutaneous tissues to expose the tibial bone, (**e**) the exposing of the tibial bone, (**f**) induction of a uni-cortical bone defect (1 mm in diameter) using an electric microdrill, (**g**) the defect hole “yellow circle,” and (**h**) closure of the dissected wound and subcutaneous tissues using Vicryl sutures.

All rats were generally anaesthetized. Induction of anesthesia was performed by intramuscular injection of 5% ketamine HCl (50 mg/kg) and 2% xylazine HCl (10 mg/kg) mixed in a ratio of 2:1 into the gluteal muscles. The anesthesia was maintained by the inhalation of isoflurane 1–2% [48]. After anesthesia, a surgical operation was performed on the rats, as presented in Figure 2, where the rats’ hind limbs were clipped, shaved, and prepared aseptically. Then, they were made recumbent laterally, exposing the tibial region medially. The skin was incised at the flat part of the tibial shaft away from the articular surface. Then dissection to subcutaneous tissues and stripping of the periosteum were performed to expose the tibial bone. Afterward, a sterilized electric microdrill (Strong, micro-drill, China) was used to induce a uni-cortical bone defect (1 mm in diameter) in the tibia of both limbs using a 1 mm drill bit in the upper cortex. The developed defects were washed with sterile saline several times. The wound, subcutaneous tissues, and skin were sutured using absorbable sutures. Postoperatively, the animals were injected with Ceftriaxone HCl^®^ (20 mg/kg) and Meloxicam^®^ (1 mg/kg) once daily for 5 days. Povidone iodine (10% *w/v*) was used to wash the wounds every day [49].

#### 2.8.3. Dosing the Formulations

Following the surgery, each rat of groups I and II received 2.5 µL (equivalent to 12.5 µg of pitavastatin and tedizolid) of D-ZIFI 1 and D-ZIFI 2, respectively. The selected formulations were injected within the defect hole of the left limb while the right limb in each rat was left as a control. At each time interval (2, 4, 6, and 8 weeks), four rats of each group were generally anesthetized by the same procedures performed in the surgery; then euthanasia was performed by the injection of thiopental sodium (90 mg/kg) intraperitoneally. After confirmation of death, the area of defect was examined visually (the sutured area was opened again to assess the progress of healing).

#### 2.8.4. Histological Assay

After euthanasia and visual examination of the defect area, the tibias and the surrounding soft tissues were separated and detached. Subsequently, the collected tibias were kept in a 10% buffered para-formaldehyde solution (10%) for 1 day and then maintained in 5% formic acid for 6 weeks to decalcify the bone tissues. Following decalcification, the tibias were rinsed with distilled water and then dehydrated using serial dilutions of different alcohols (methanol and ethanol). The collected samples were cleaned using xylene and then were fixed in hot paraffin pre-kept in a hot air oven maintained at 56 °C for 24 h. A sledge microtome was used to cut paraffin beeswax tissue blocks into 4 µm thick sections. After that, the sections were added on glass slides, de-paraffinized, and dyed with hematoxylin and eosin stain to be examined via a light electric microscope (CX21Olympusmicroscope, Tokyo, Japan) [50].

## 3. Results and Discussion

### 3.1. Preparation of Zein In Situ Forming Implants (ZIFIs)

When this system is included in an aqueous medium, solvent exchange occurs, leading to the precipitation of the polymer due to the sol–gel transformation, resulting inthe formation of an implant that fills the void space in the fractured bone [51]. The formed implants could provide sustained drug release, offering long-lasting therapeutic action depending on the concentration of the used polymer. Zein is a suitable candidate for the formation of the IFIs via the solvent-induced phase inversion technique, as it precipitates easily in aqueous medium due to its inherent hydrophobicity [52]. The selection of DMSO as a solvent was done for many reasons: it possesses low systemic toxicity; being organic in nature enables the dissolution of both zein as well as the added drugs; furthermore, it is a polar water-miscible solvent with high water affinity, which allows for fast polymer precipitation. Moreover, DMSO has low viscosity, so a high amount of polymer and drug can be dissolved without negatively affecting the injectability of the formulation [51].

### 3.2. In Vitro Characterization of the Prepared Zein In Situ Forming Implants (ZIFIs)

#### 3.2.1. In Vitro Solidification Time

The solidification time of IFIs is considered one of the most crucial factors. The injected solution needs to solidify in a reasonable time after injection to avoid an extensive burst drug release. Table 1 shows the solidification time values of the tested ZIFIs. From the table, it is clear that by increasing zein concentration, the solidification time significantly decreased (*p* < 0.05). Formulation ZIFI 1, composed of the lowest zein concentration (10% *w*/*v*), solidified after 104.9 ± 4.9 s, whereas formulation ZIFI 3, formed using the highest zein concentration, solidified after 48.9 ± 3.6 s. This could be attributed to the increased hydrophobicity of the system by increasing zein concentration and the consequent faster precipitation. The relatively more hydrophilic system (10% *w*/*v* zein) could endure the outflux of DMSO from its solution and the influx of the buffer for a certain time, while the hydrophobic ones immediately precipitate upon contact with the buffer. Generally, polymers dissolved in DMSO tend to precipitate relatively faster than other less miscible solvents (e.g., triacetin, ethyl benzoate) due to the high water miscibility of DMSO, allowing fast water influx through the system and, consequently, a rapid rise in precipitation [53]. These findings are in agreement with Elkasabgy et al. [36].

#### 3.2.2. Rheological Properties

The rheological behavior of the injectable formulations is critical. Systems with high viscosity values show poor injectability, necessitating large injection forces. The rheological behavior of liquid formulations could be divided into Newtonian and non-Newtonian flow depending on the change in viscosity as per the change in shear rate values. Systems having constant viscosity values are known to be Newtonian, while those with variable viscosity values are called non-Newtonian. Non-Newtonian flow could be shear thinning or shear thickening [54].

According to Farrow’s constant (*N*), it is obvious that both formulations ZIFI 1 and 2, made up from 10 and 20% *w*/*v* zein, respectively, showed Newtonian behavior where the obtained N values were approximately 1. In contrast, formulation ZIFI 3, fabricated using the highest zein concentration (30% *w*/*v*), possessed an *N* value of 1.321, indicating the shear-thinning behavior of the system. The difference in the obtained flow behaviors indicates the dependence of viscosity on the concentration of the used polymer [55]. In simple words, this can be attributed to the increased extent of entanglements between the polymer chains at high concentrations. In concentrated solutions, the interconnection between polymer chains is in a dynamic state; that is, the disrupted entanglements caused by low shear rate values are replaced by others, so a slight change in viscosity is detected initially. However, by increasing the shear rate values, the disrupted entanglements cannot be replaced totally with new ones at the same time, resulting in the viscosity gradually decreasing (shear-thinning behavior). Shear-thinning flow is advantageous in the parenteral field, as the liquid viscosity decreases on being shaken before injection, so improved injectability could be attained [56].

All formulations showed reasonable viscosity values, where the obtained values for ZIFI 1 and 2 were 22.4 ± 0.7 and 66.6 ± 34.5 cP, respectively. In contrast, the viscosity values at the minimum and maximum shear rates for formulation ZIFI 3 were 300.84 and 108.83 cP, in that order. As observed, increasing the concentration of zein resulted in a significant increase (*p* < 0.05) in the viscosity of the formulations. Again, this could be justified by expanding the extent of polymer entanglements, which in turn restricted the movement of individual chains [11].

#### 3.2.3. Injectability Test

The injectability of parenteral solutions is an essential parameter to be considered during the formulation. The flow rate of the tested formulations (ZIFI 1, ZIFI 2, and ZIFI 3) ranged from 8.6 ± 3.1 to 1.6 ± 0.1 mL/min (Table 1). Results reveal the significant effect of zein concentration on the flow rate values, where the flow rate of the formulation was slowed down on increasing the zein concentration. This might be simply justified by the increased viscosity of the solution imparted by the increased zein concentration. However, the obtained flow rate values of the three tested concentrations were less than that of the commercial product Betolvex^®^ (0.3 ± 0.1 mL/min), indicating reasonable and acceptable injectability of all the prepared formulations.

#### 3.2.4. In Vitro Release Study

**Figure 3:**
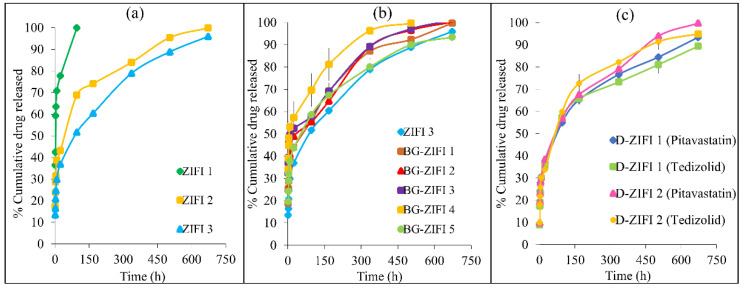
In vitro release profiles of pitavastatin from formulations (**a**) ZIFI 1, 2, and 3.(**b**) BG-ZIFI 1, 2, 3, 4, and 5, in comparison with ZIFI 3. (**c**) In vitro release profiles of pitavastatin and tedizolid from D-ZIFI 1 and D-ZIFI 2.

In vitro drug release elaborates the effect of formulation factors. Besides, it could predict the in vivo behavior of the examined formulations. Most of the drug (≈100%) was released from its PBS suspension within 4 h, signifying the appropriateness of the release procedures as well as the used dialysis membrane. The release profiles of pitavastatin from the ZIFIs are represented in Figure 3a. All the investigated formulations (ZIFI 1, ZIFI 2, and ZIFI 3) suffered from initial fast drug release, after 24 h (Q_24h_) (Table 1). This burst release might be ascribed to the release of drug attached to the surface of the formed implant. Additionally, the formation of minimal pores during the evolution of DMSO from the zein matrix to the aqueous medium might be a potential cause for the initial burst release [57,58,59].

In addition, one notices that increasing zein concentration had a significant lowering effect (*p* < 0.05) on burst drug release within the first 24 h (Q_24h_), where formulation ZIFI 3 possessed the lowest burst release, of 36.9 ± 0.40%, compared to formulations ZIFI 1 and 2, which had Q_24h_ values of 77.70 ± 0.58% and 43.15 ± 0.00%, respectively. This reduction in burst drug release associated with increasing zein concentration could be attributed to the faster solidification of the formed implant. Indeed, a shorter solidification time avoided missing part of the loaded dose and enhanced the entrapment of most of the drug inside the formed matrix [60]. The Q_24h_ results are in good compliance with the solidification time results. The initial burst release was followed by a slow drug release phase, which continued up to 4 days in the case of ZIFI 1, while ZIFI 2 and ZIFI 3 showed a sustained release for 28 days. This slow-release phase might be ascribed to the diffusion facilitated release of the drug molecules present in the matrix [51].

The release data best fitted the Korsmeyer–Peppas model with the highest correlation coefficient values (r). As shown in Table 1, the release rate constant (k) values depend on zein concentration, where a significant decline (*p* < 0.05) in k values was observed with increasing zein concentration. Again, slow drug release associated with higher zein concentrations might be linked to the faster solidification as well as the formation of a denser and more compact matrix with enhanced system hydrophobicity. This resulted in the formation of fewer water channels, consequent to which the implant could withstand the slow diffusion of water and subsequent slower matrix erosion.

From the obtained release results, it could be concluded that formulation ZIFI 3, made up of the highest zein concentration (30% *w*/*v*), possessed the most sustained drug release profile with the least initial burst release. Hence, ZIFI 3 was chosen for further improvements. 

### 3.3. Preparation and Evaluation of Zein In Situ Forming Implants Loaded with Bioactive Glass Nanoparticles (BG-ZIFI)

#### 3.3.1. Evaluation of Bioactive Glass Nanoparticles by Transmission Electron Microscope (TEM)

**Figure 4:**
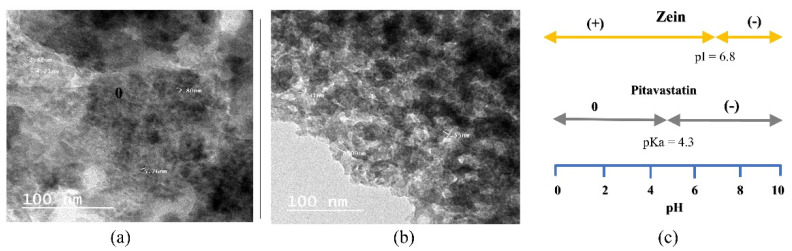
TEM micrographs of (**a**) non-doped BG and (**b**) BGT_5_. (**c**) A representation of the electrostatic interaction between zein and pitavastatin.

As shown in Figure 4, doped and non-doped BGs were found to be on the nanoscale. Both types showed a typical agglomerated appearance of BG nanoparticles [61]. BGT_0_ showed a smaller size (2.47–5.76 nm) than BGT_5_ (6.31–8.09 nm). It is suggested that titanium acts as a network modifier leading to double condensation and a consequent increase in the particle size [42].

#### 3.3.2. In Vitro Release Study

ZIFIs augmented with BG were obtained in the attempt to improve the bioactivity of the prepared implants [62]. The release profiles of the tested implants after loading with BG are illustrated in Figure 3b. By comparing the Q_24h_ values of formulations BG-ZIFI 1, 2, 3, and 4 loaded with 1, 3, 5, and 10% *w*/*v* BGT_0_, respectively, with that of ZIFI 3 lacking BG addition, it was observed that surprisingly the Q_24h_ increased significantly (*p* < 0.05) with the four concentrations of BGT_0_ (Table 1). Moreover, it was noticed that this increase is directly proportional (*p* < 0.05) to the concentration of the added BG.

By analyzing the release rate constants (k), based on the Korsmeyer–Peppas model (Table 1), it was noticed that faster release occurred after the addition of non-doped BG compared to formulation ZIFI 3 (*p* < 0.05). Moreover, formulation BG-ZIFI 4 (loaded with the highest concentration of BGT_0_, 10%) showed the highest k value (*p* < 0.05). This finding might be linked to the rise in the pH of the fluid surrounding the formed implant after mixing with PBS, which in turn results in boosting the solubilization of zein protein (soluble in an alkaline medium), with a resulting increased water diffusion and matrix degradation [63]. The rise in pH was not detected with ZIFI 3, where the pH value ranged between 6.93 and 6.96, while the pH measured after adding the BGT_0_ ranged from 7.63 and 9.25. Additionally, it was noticed that pH values increased significantly (*p* < 0.05) with increasing BGT_0_ concentration. The elevation in pH might be linked to the alkalinity of the used glass ingredients.

Another key factor influencing the pH dependence of the release data is the electrostatic interaction between zein and pitavastatin. As represented in Figure 4c, zein is an amphoteric protein carrying a net positive charge at a pH lower than its isoelectric point (pI; 6.8) and a net negative charge above its pI [64]. Pitavastatin is a weakly acidic drug with a pKa value of 4.1, so it carries a net negative charge above its pKa [65]. At alkaline pH values above 7, both zein and pitavastatin carried negative charges and, hence, electrostatic repulsion occurred, resulting in higher release rates. These results are in agreement with Bouman et al. [64]. Moreover, the hydrophilic nature of BG imparts certain hydrophilicity to the implants, leading to a faster drug release [66].

From these results, it could be concluded that formulation BG-ZIFI 1, loaded with 1% *w/v* BGT_0_, showed the most sustained drug release profile. Hence, the same concentration of BGT_5_ was selected for further optimizations to improve the bioactivity of the implant. Titanium is known to improve the mechanical properties of the implant [67], enhance osseointegration [68], and impart antibacterial activity [69].

As shown in Figure 3b, drug release from the implant prepared with BGT_5_ (BG-ZIFI 5) showed a comparable release rate constant and Q_24h_ to those of ZIFI 3. However, by comparing the IFIs prepared using the same concentration (1% *w*/*v*) of both BGT_0_ and BGT_5_, it was manifested that the implant loaded with BGT_5_ showed significantly less Q_24h_ and release rate values (*p* < 0.05). This might be attributed to the neutralization of the alkaline pH of the glass nanoparticles by the inherent acidity of TiO_2_ in the BGT_5_ structure [70]. Another potential reason for the sustainment of a BGT_5_-loaded implant could be attributed to the enhanced mechanical strength, which might decrease the degradation rate of the implant [67].

From the obtained results, formulation BG-ZIFI 5 loaded with 1% BGT_5_ was selected for further modifications based on its sustained release behavior.

### 3.4. Preparation of Dual-Medicated Zein In Situ Forming Implant (D-ZIFI)

Multi-active drug delivery systems present an excellent solution for combination therapy. Dual-medicated implants loaded with tedizolid in addition to pitavastatin were fabricated to enhance the bone healing process. As an approach to potentiate the therapeutic usefulness of the prepared implants, tedizolid phosphate was added to the chosen implant (BG-ZIFI 5) to treat associated infections [71]. It is a potent antibacterial pro-drug that is approved for its efficacy against methicillin-resistant *staphylococcus aureus* and acts by inhibiting the bacterial protein synthesis via binding to the 50S ribosomal subunit [72,73]. It is activated in vivo to its active metabolite (tedizolid) by the action of esterase enzymes such as alkaline phosphatase (ALP), which is produced naturally during the bone formation process [74].

Furthermore, a porogenic agent was added to improve the porosity of the prepared implant (D-ZIFI 1). Sodium hyaluronate was chosen as the porogenic agent [75]. It is a derivative of hyaluronic acid, a naturally produced high-molecular-weight non-sulfated glycosaminoglycans composed of D-glucuronic acid and N-acetyl glucosamine disaccharide units. It is a biocompatible biomacromolecule that has been broadly used in bone tissue engineering [76,77]. Formulation D-ZIFI 2 lacking the addition of both BGT_5_ and sodium hyaluronate was prepared for comparison purposes.

### 3.5. Characterization of Dual-Medicated Zein In Situ Forming Implant (D-ZIFI)

#### 3.5.1. In Vitro Solidification Time

The tested formulations (D-ZIFI 1 and 2) solidified in less than 1 min, which assured their suitability (Table 1). D-ZIFI 1 showed a slower solidification time (*p* < 0.05) than formula D-ZIFI 2, which might be due to the increased hydrophilicity imparted by the addition of sodium hyaluronate and BGT_5_.

#### 3.5.2. Rheological Properties

Based on Farrow’s constant (*N*), both formulations D-ZIFI 1 and 2 showed shear-thinning behavior where the calculated N values were 1.23 and 1.15, respectively. This might be explained as mentioned in Section 3.2.2.

#### 3.5.3. Injectability Test

Both formulations (D-ZIFI 1 and 2) showed reasonable injectability, which was less than that of the marketed product Betolvex^®^ (Table 1). Additionally, and compared to the unmodified formulation ZIFI 3, it was manifested that D-ZIFI 1 possesses a lower flow rate value (*p* < 0.05). This might be assigned to the extra added components. By investigating the difference between D-ZIFI 1 and 2, it was shown that formulation D-ZIFI 1 possesses a slower flow rate, which could be attributed to its increased viscosity.

#### 3.5.4. In Vitro Release Study

The second derivative method was used to resolve the problem of overlapping spectra of pitavastatin and tedizolid, as well as to allow the simultaneous determination of both without the need for prior separation [78].

The release data and graphs of formulations D-ZIFI 1 and 2 are compiled in Table 1 and Figure 3c. Regarding formulation D-ZIFI 1, it is manifested that both drugs showed a sustained release profile with Q_24h_ and release rate constant values of 38.47 ± 0.74% and 14.29 ± 0.52 h^−1^ versus 33.77 ± 0.54% and 13.34 ± 0.20 h^−1^ for pitavastatin and tedizolid, respectively. Pitavastatin showed a release pattern similar to that of formulation BG-ZIFI 5 lacking the addition of tedizolid and porogenic agent, indicating the lack of interference with the drug release.

Formulation D-ZIFI 2 showed in vitro results comparable to those of D-ZIFI 1. Both showed sustained release behavior with suitable solidification time and acceptable flow rates, so they were selected for further investigations and in vivo study on rats.

#### 3.5.5. Fourier-Transform Infrared (FTIR) Spectroscopy

**Figure 5:**
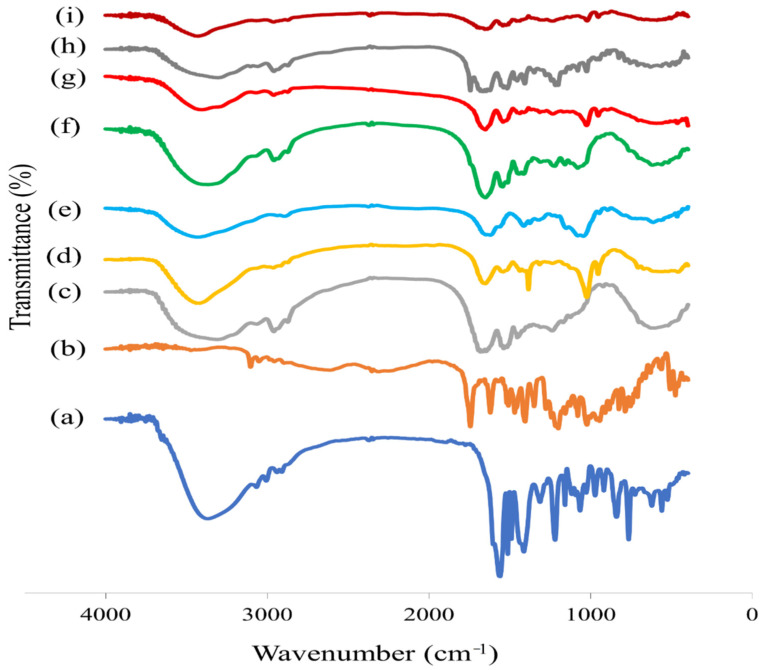
The IR charts of (**a**) pitavastatin, (**b**) tedizolid, (**c**) zein, (**d**) BGT_5_, (**e**) sodium hyaluronate, (**f**) physical mixture of D-ZIFI 1, (**g**) the implant of D-ZIFI 1, (**h**) physical mixture of D-ZIFI 2, and (**i**) implant of D-ZIFI 2.

FTIR spectra of D-ZIFI 1, D-ZIFI 2, their physical mixtures, as well as their separate components are shown in Figure 5. The FTIR spectrum of pitavastatin shows several characteristic bands; it demonstrates a distinguishable stretching band at 3363.86 cm^−1^ for O–H stretching and peaks at 3066.82, 1585.48, and 1558.48 cm^−1^ for C–H aromatic stretching, C=O stretching, and C=C aromatic stretching, respectively. A peak at 1026.13 cm^−1^ for C–F alkyl halide stretching is also observed. The spectrum is in line with the literature [79]. Tedizolid reveals its characteristic bands due to C–H, C=C, and C=O stretching vibrations at 3105.39, 1743.65, and 1620.21 cm^−1^, respectively. It also shows a peak at 1022.27 cm^−1^ due to C–F stretching vibration [80]. The spectrum of zein shows the N–H stretching band of amides at 3305.99 cm^−1^ besides its protein characteristic peaks of amide I and amide II at 1631.78 and 1539.20 cm^−1^, respectively [81].

Regarding the FTIR spectrum of BGT_5_, the presence of -OH stretching and bending at 3441.01 and 1635.64 cm^−1^ might be due to the presence of surface water molecules. It shows characteristic peaks for P–O stretching and bending at 1041.56 and 447.493 cm^−1^, respectively. It also reveals bands for the Si–O–Si stretching of non-bridging oxygen atoms and symmetric stretching of bridging oxygen between tetrahedral at 871.82 and 775.38 cm^−1^, respectively [82,83]. These bands confirm the presence of SiO_2_, the main component of the glass system, as the network former. The spectra additionally show a characteristic peak located at 713.66 cm^−1^, indicating Ti–O vibration [84,85]. The sodium hyaluronate spectrum reveals a band at 3417.86 cm^−1^ corresponding to the overlapping of -OH and -NH groups and at 2893.22 cm^−1^ for aliphatic -CH stretching, in addition to -CH stretching and bending at 1620.21 and 1415.77 cm^−1^, respectively [86].

The spectra of both D-ZIFI 1 and 2 physical mixtures lacked interaction between any of them, as the specific functional groups of the two drugs, as well as the used ingredients, were present. However, with a minor shift in their positions and a slight decrease in the intensity, the spectrum of both D-ZIFI 1 and 2 presented a shift and a sharp decline in the intensity of -OH and -NH bands, indicating the possible hydrogen bond formation between the NH groups of zein with pitavastatin (-OH groups) and/or sodium hyaluronate (-OH and -NH groups).

#### 3.5.6. Differential Scanning Calorimetry (DSC)

**Figure 6:**
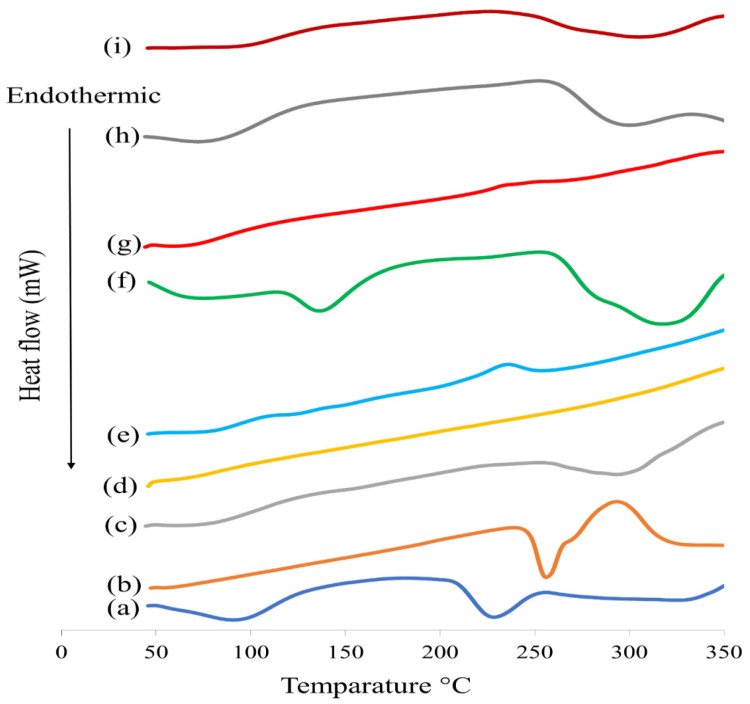
DSC thermograms of (**a**) pitavastatin, (**b**) tedizolid, (**c**) zein, (**d**) BGT_5_, (**e**) sodium hyaluronate, (**f**) physical mixture of D-ZIFI 1, (**g**) the implant of D-ZIFI 1, (**h**) physical mixture of D-ZIFI 2, and (**i**) implant of D-ZIFI 2.

DSC thermograms of D-ZIFI 1, D-ZIFI 2, their physical mixtures, and separate components are illustrated in Figure 6. The pitavastatin spectrum demonstrates an endothermic peak at 95 °C, which might be due to the evaporation of associated water molecules [87], and at 228.3 °C, corresponding to its melting temperature, indicating its crystallinity [88]. For tedizolid, its thermogram reveals an endothermic peak at 256.17 °C, relating to the melting of the crystalline form [89]. Concerning the thermal behavior of zein, a broad endothermic band at 79.1 °C due to protein denaturation through the breaking up of hydrogen bonds, disulfide bridges, and hydrophobic interactions can be observed and is consistent with the literature [90]. Another endothermic peak is detected at 293.2 °C, corresponding to its melting point [91]. Sodium hyaluronate exhibits an exothermic peak at 243.2 °C, corresponding to its thermal decomposition [92].

The thermograms of D-ZIFI 1 and 2 physical mixtures show complete disappearance of pitavastatin and tedizolid peaks. This disappearance could be ascribed to the molecular dispersion of both drugs inside the implant components during the heating procedures, resulting in the dilution of drugs in the formed mixture. Both thermograms display the melting peaks of zein. In the case of formulation D-ZIFI 1, zein’s melting peak completely diminishes while a marked decrease in its intensity can be observed with formulation D-ZIFI 2. Again, the diminishing of drugs’ peaks along with the vanishing of any characterizing peak for the other components in formulation D-ZIFI 1 might signify the development of a new matrix system.

#### 3.5.7. Morphological Examination 

**Figure 7:**
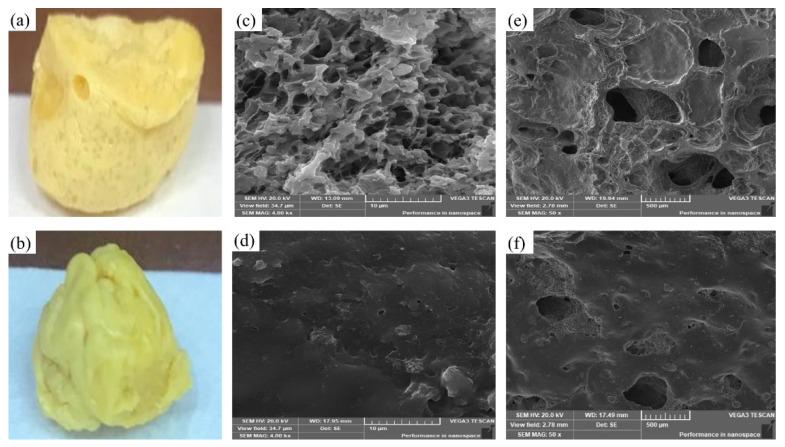
Photographs of (**a**) D-ZIFI 1 and (**b**) D-ZIFI 2 implants. SEM images of the cross-sectional morphologies of (**c**) D-ZIFI 1 and (**d**) D-ZIFI 2 after immersion in PBS for 1 day and (**e**) D-ZIFI 1 and (**f**) D-ZIFI 2 after immersion in PBS for 7 days.

To assess the effect of the added BGT_5_ and porogenic agent, the external and internal morphologies of formulations D-ZIFI 1 and 2 were studied. On examining the external morphology, the surface of air-dried D-ZIFI 1 one day post-formation shows a smooth surface with visible pores (Figure 7a), while that of D-ZIFI 2 is characterized by an irregular, non-porous surface (Figure 7b).

The cross-sectional micrographs of both formulations were also compared after 1 and 7 days after implant formation. It can be observed that formulation D-ZIFI 1 shows a porous internal structure after 1 day (Figure 7c), and an enhanced formation of pores is detected after 7 days (Figure 7e). In contrast, formulation D-ZIFI 2 lacking the addition of BGT_5_ and the porogenic agent shows a non-porous internal structure after 1 day (Figure 7d). Some pores began to form after 7 days (Figure 7f) but to a lesser extent compared to formulation D-ZIFI 1. In general, pores are formed due to the solvent exchange process of DMSO into the aqueous medium. Pore formation is enhanced with passage of time due to the gradual degradation of the formed matrix and the drug release from the implant. Regarding formulation D-ZIFI 1 loaded with BG and the porogenic agent, the more enhanced porosity detected could be attributed to the presence of sodium hyaluronate in the formulation, which acts as a water-soluble porogenic agent forming interconnected pores during its dissolution in the aqueous medium [93]. Moreover, possible dissolution of some of the BGT_5_ nanoparticles might occur during the leaching process, leading to more pore formation [60]. 

#### 3.5.8. Effect of Gamma Sterilization

On comparing D-ZIFI 1 and 2 before and after sterilization, no significant difference (*p* > 0.05) in the in vitro solidification time and flow rate could be detected. The in vitro release profiles of the formulations were similar before and after sterilization and were superimposed. The calculated similarity factor (*f*_2_) values for pitavastatin and tedizolid in both formulations were >50, signifying similar release profiles. Gamma sterilization is used for the sterilization of several compounds without influencing their physicochemical properties [94] and is approved as a suitable method for the sterilization of the examined implants [46].

### 3.6. In Vivo Animal Study

Employing an animal model during the pre-clinical phase is a key step for assessing the efficacy of new formulations [95]. The normal bone healing process after any fracture includes three stages: inflammatory, reparative, and remodeling stages. The inflammatory stage begins instantly after bone damage. Inflammation and formation of a hematoma at the fracture site characterize this phase. Before the end of the inflammatory stage, the reparative stage starts. This stage is characterized by the formation of fibroblasts, chondroblasts, and osteoblasts. Additionally, the formation of callus tissue in and around the fracture site occurs. This callus tissue is composed of blood vessels, cartilage, fibrous connective tissues, woven bone, and osteoid. The fibrocartilaginous (soft) callus develops in time into a bony (hard) callus. Afterward, the remodeling stage starts, which includes the replacement of the fibrous woven bone with strong lamellar bone along with the resorption of additional callus. This stage is a long one and may take from several months up to many years.

This study aimed at monitoring the healing rate of an induced fracture by observing callus formation. The effect of D-ZIFI 1 and 2 on the healing process of the induced defects in the left limbs of rats was compared with the untreated defects in the right limbs by macroscopical and microscopical examination of the defect site at the 2nd, 4th, 6th, and 8th week post-surgery.

Initially, D-ZIFI 1 displayed a great hemostatic effect, comparable to that expected by the usage of bone wax (a product used to provide hemostasis in a bleeding bone [96]). This remarkable finding could be attributed to the hemostatic effect of sodium hyaluronate as stated by Cho et al. [97].

**Figure 8:**
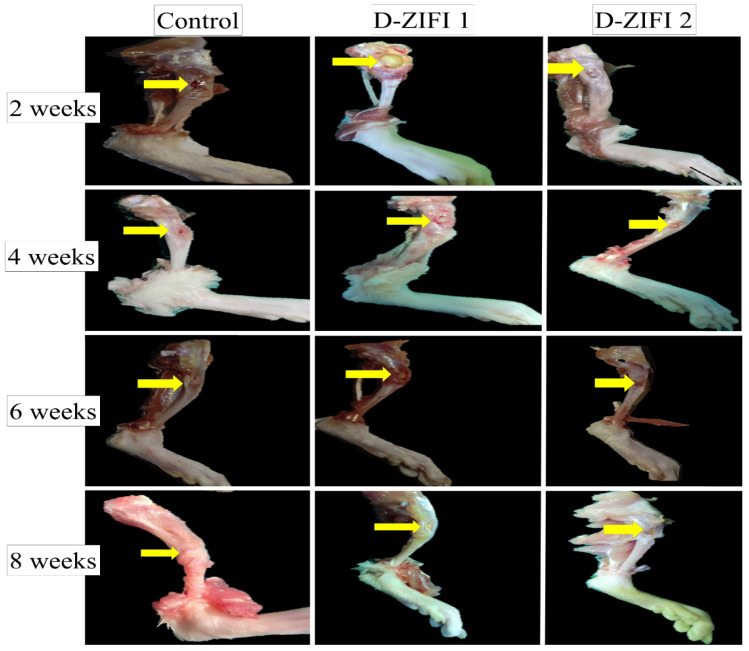
Photographs of a rat’s tibia in the control limb and after injecting formulations D-ZIFI 1 and D-ZIFI at the 2nd, 4th, 6th, and 8th week post-surgery.

Macroscopical examination of the collected tibias at different observation periods revealed that in all cases no signs of infections, fractures, or osteolytic reaction occurred (Figure 8). At 2nd week post-surgery, the defect area in the control group showed a reddish spot filled with organized hematoma and fibrous tissues adhesions. In contrast, the injected implant in the D-ZIFI 1 group was seen plugging and occupying the induced defect. In addition, it was enclosed with fibrous connective tissue, while in the D-ZIFI 2 group, the non-porous compact implant filled the defect with surrounding reddish fibrous connective tissue.

At the 4th week post-surgery, the induced defect in the control group did not show a significant difference compared to the 2 weeks post-surgery point. In the case of the D-ZIFI 1 group, the implant observed within the bone defect was smaller than the previous one, indicating ongoing biodegradation. Besides, it was bulged and surrounded by a reddish vascularized zone. However, in the case of the D-ZIFI 2 group, the implant was still detected within the defect but had decreased in size with increased surrounded fibrous tissue.

At the 6th week post-surgery, the bone defect was still clear in the control group but showed whitish hard connective tissue. In the case of the D-ZIFI 1 group, the implant decreased more in size compared to the previous time point but was still bulging above the bone surface and surrounded with fibrous tissue adhesions. On the other side, in the D-ZIFI 2 group, the implant was completely enclosed and masked with reddish fibrous connective tissue filling the bone defect.

At the 8th week post-surgery, the bone defect had almost disappeared and was covered with organized fibrous connective tissue with hard adhesions in the control group. However, in the D-ZIFI 1 group, the bone defect hole decreased significantly in size and its center was occupied by small remnants of the implant. In the D-ZIFI 2 group, the implant nearly disappeared and the defect was surrounded by hemorrhagic fibrous connective tissues.

It could be concluded that formulations D-ZIFI 1 and 2 degraded over time due to creeping substitution and the formation of new bony tissues parallel to the degradation of the implant. This confirmed the success of the prepared implant concerning its biodegradability and osteoconductivity. The obtained findings have not clearly displayed the healing steps and the nature of the formed tissues around the defect. Consequently, further histological examination was performed.

#### Histological Assay

**Figure 9:**
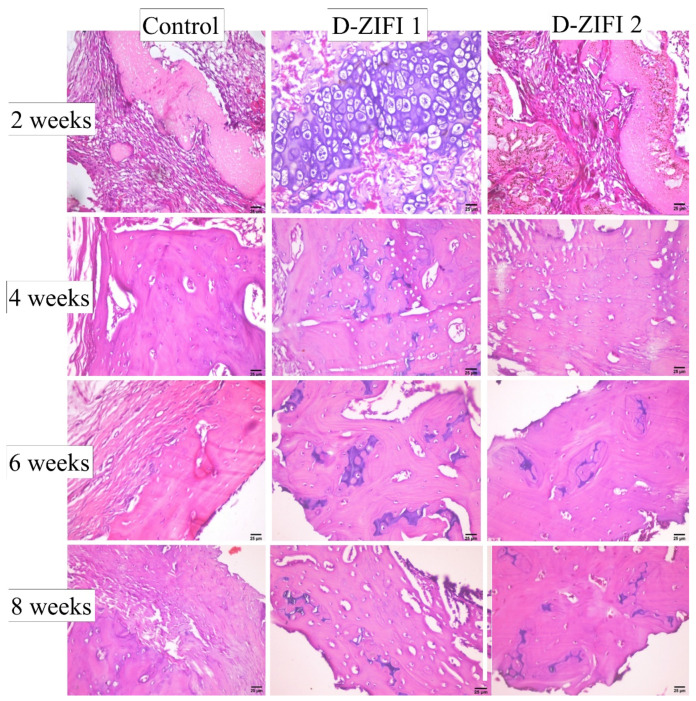
Histological sections of punctures in the tibia of rats stained with hematoxylin and eosin at magnification 40X of non-treated tibial puncture, tibial puncture treated with D-ZIFI 1, and tibial puncture treated with D-ZIFI 2 after 2, 4, 6, and 8 weeks of treatment.

Microscopical examination of tissue sections of the bone defect area collected in successive observation periods was performed (Figure 9). At the 2nd week post-surgery, the defect area was filled with well-organized fibrous connective tissue with distinct boundaries between the two lamellar bone and the cortical bone in both control and D-ZIFI 2 groups. However, in the D-ZIFI 1 group, there was hypertrophy of chondrocytes with the migration of woven bone to replace it at the cartilage bone junction. In harmony with the formerly stated normal phases of bone healing, the obtained results indicate active ongoing reparative phase and suggest soft callus formation, which demonstrates rapid bone healing.

At the 4th week post-surgery, no significant variation could be observed with the control. In the case of the D-ZIFI 1 group, the defect area was filled with a newly formed bone containing a hyaline-like matrix surrounded by mature osteocytes, accompanied with progressive resorption of the formed callus, declaring active bone formation. However, the D-ZIFI 2 group showed that the defect area was still occupied by fibrous connective tissue containing numerous mature osteocytes.

At the 6th week post-surgery, no change was detected with the control group. A small area of the woven bone surrounded by osteoblasts and newly formed bone filled with mature osteocytes in a hyaline-like matrix was observed in the D-ZIFI 1 group. However, in D-ZIFI 2, woven bone surrounded by numerous osteoblasts and a few osteocytes were visible in the investigated section, with abnormal newly formed bone containing small amounts of a hyaline-like matrix.

At the 8th week post-surgery, newly formed blood vessels were observed spreading through the well-organized fibrous connective tissue in the control group. In D-ZIFI 1 and D-ZIFI 2, the formation of new bone containing small amounts of a hyaline-like matrix surrounded by osteoblasts and osteocytes was enhanced compared to the previous time point. Additionally, fibrous tissue reactivity was observed in the D-ZIFI 2 group.

These findings suggest the slow, improper bone healing of the untreated punctured area in the control group. The D-ZIFI 2 group showed delayed, less efficient bone healing compared to the D-ZIFI 1 group. This study validated the success of formulation D-ZIFI 1 in providing accelerated bone healing due to the synergistic effect of its ingredients. Pitavastatin enhances the osteoblastic activity by several mechanisms, as mentioned previously. It induces the production of several vascular endothelial growth factors and stimulates BMP-2 and osteocalcin production as mentioned elsewhere [98].

Sodium hyaluronate had a great impact on the success of the formulation by being a porogenic agent. The formed interconnected porous structure aided in the osseointegration of the implant with the fractured bone. It facilitated cell growth by permitting the transportation of nutrients and metabolic wastes formed during the bone healing process through the formed implants. The formed implants acted as a pathway for cell migration and proliferation. In addition, porosity might aid in the vascularization (the formation of new blood vessels) throughout the engineered tissue [99,100,101]. Moreover, sodium hyaluronate might promote the formation of hydroxyapatite on the surface of the formed implants, enhance ALP activity, and promote osteogenic-differentiation-related protein expression. Furthermore, it plays a key role in maintaining the growth factors within the affected tissue and enhances the osteogenic effect of BMP-2, besides being a ligand for the cluster of differentiation 44, a transmembrane receptor expressed by many cells to stimulate differentiation, migration, and vascularization of endothelial cells [102,103].

BGT_5_ enhanced the bioactivity of the implant as BG combines two beneficial characteristics, being osteoinductive and being osteoconductive, inducing osteogenesis by stimulating the differentiation of cells toward osteoblast formation [104]. BGT_5_ might create a permanent bond to bone via osseointegration, so it enhances cell attachment and proliferation. In addition, it acts as a support onto which bone cells can grow and proliferate [105,106]. The addition of titanium improves the mechanical properties of the formed implants by its characteristic load-bearing support ability, showing mechanical properties simulating natural bone tissues [67]. Additionally, titanium possesses antibacterial effects via the production of reactive oxygen species (ROS), which affects the bacterial cells by different mechanisms, leading to their death [107,108]. This antibacterial effect might aid in the bone healing process [69].

The enhanced therapeutic effect of formulation D-ZIFI 1 might be ascribed to the use of multi-active implants loaded with both pitavastatin and tedizolid phosphate. Being an antibiotic, tedizolid phosphate can control and treat the bacterial infections associated with bone injuries and, in turn, can speed up the bone formation process. The in situ local application of the antibiotic might decrease the need for prolonged oral or injected antibiotic courses required for bone infections associated with injuries [109].

## 4. Conclusions

This research is the first to formulate zein protein as an injectable in situ forming implant for the healing of bone defects as a promising alternative to surgical interventions in the healing of bone injuries. Zein in situ forming implants were successfully prepared using a solvent-induced phase inversion method. Preparing implants with various zein concentrations revealed that increasing the concentration of zein leads to faster solidification, a lower flow rate, and more sustained drug release. The highest concentration zein (30% *w*/*v*)-based implants were modified by being loaded with different concentrations of non-doped bioactive glass, where a faster drug release pattern was detected on increasing the bioactive glass concentration. Titanium-doped bioactive glass was added to improve the osseointegration and mechanical properties of the implant. Dual-medicated zein in situ forming implants were prepared by the co-addition of an antibiotic (tedizolid) together with pitavastatin to the implants loaded with titanium-doped bioactive glass. Further optimization of the dual-medicated zein in situ implants was carried by the addition of a porogenic agent (sodium hyaluronate). The optimized dual-medicated implant showed rapid solidification, reasonable injectability, sustained drug release up to 28 days, and porous microstructure. In vivo studies on surgically induced bone defects on Sprague–Dawley rats confirmed the success of the optimized dual-medicated zein in situ forming implant in accelerating proper bone healing in rats’ tibia.

Although this study has come a long way in the application of zein as an implant matrix for bone tissue engineering, further future research to optimize the fabricated implants is still required. The use of zein-based implants for the treatment of large-sized bone defects requires more attention and extra studies. Moreover, scaling-up of the fabricated implants should be taken into consideration.

## Figures and Tables

**Table 1:** Composition and characterization of zein in situ forming implants.

Formulation Code ^a^	Composition				Characterization			
	Zein (% *w*/*v*)	BGT_0_ (% *w*/*v*)	BGT_5_ (% *w*/*v*)	Sodium Hyaluronate (% *w*/*v*)	Solidification Time (s)	Flow Rate (mL/min)	Q_24h_ (%)	K (h^−1^)
**ZIFI 1 ^a^**	10	---	---	---	104.9 ± 4.9	8.6 ± 3.1	77.70 ± 0.58	39.86 ± 0.03
**ZIFI 2 ^a^**	20	---	---	---	65.9 ± 2.5	2.9 ± 0.4	43.15 ± 0.00	19.97 ± 0.17
**ZIFI 3 ^a^**	30	---	---	---	48.9 ± 3.6	1.6 ± 0.1	36.9 ± 0.40	14.23 ± 0.94
**BG-ZIFI 1 ^a^**	30	1	0	---	---	---	43.94 ± 0.50	21.94 ± 0.37
**BG-ZIFI 2 ^a^**	30	3	0	---	---	---	48.88 ± 1.11	28.64 ± 1.46
**BG-ZIFI 3 ^a^**	30	5	0	---	---	---	52.47 ± 0.69	34.76 ± 0.21
**BG-ZIFI 4 ^a^**	30	10	0	---	---	---	57.27 ± 0.53	36.62 ± 0.20
**BG-ZIFI 5 ^a^**	30	0	1	---	---	---	38.84 ± 0.53	15.78 ± 0.70
**D-ZIFI 1 ^b^**	30	0	1	3	52.4 ± 3.1	1.2 ± 0.1	38.47 ± 0.74 ^c^	14.29 ± 0.52 ^c^
							33.77 ± 0.54 ^d^	13.34 ± 0.20 ^d^
**D-ZIFI 2 ^b^**	30	0	0	---	43.9 ± 2.5	1.5 ± 0.1	36.5 ± 0.90 ^c^	13.85 ± 0.40 ^c^
							35.28 ± 1.08 ^d^	12.42 ± 1.07 ^d^

^a^ Prepared in situ forming implants contained 5 mg of pitavastatin. **^b^** Prepared in situ forming implants contained 5 mg of pitavastatin and 5 mg of tedizolid. ^c^ Drug release data of pitavastatin from the in situ forming implant. ^d^ Drug release data of tedizolid from the in situ forming implant. Data are presented as the mean ± SD (*n* = 3). **Abbreviations: BGT_0_**, non-doped bioactive glass nanoparticles; **BGT_5_**, titanium-doped bioactive glass nanoparticles; **ZIFI**, zein in situ forming implant; **BG-ZIFI**, zein in situ forming implant augmented with bioactive glass nanoparticles; **D-ZIFI 1**, optimized dual-medicated zein in situ forming implant; **D-ZIFI 2**, non-optimized dual-medicated zein in situ forming implant; **Q_24h_**, percentage of drug released after 24 h; **k**, release rate constant.

## Data Availability

The data presented in this study are available in the article.
